# Testing network clustering algorithms with natural language processing

**DOI:** 10.3389/frai.2025.1635436

**Published:** 2025-11-13

**Authors:** Ixandra Achitouv, David Chavalarias, Bruno Gaume

**Affiliations:** 1 Sorbonne University, CNRS, LIP6, Paris, France; 2 Complex Systems Institute of Paris île-de-France (ISC-PIF, UAR3611), Paris, France; 3 Centre d'Analyse et de Mathématique Sociales (CAMS, UMR8557), Paris, France; 4 Cognition, Langues, Langage, Ergonomie (CLLE, UMR 5263), CNRS, Paris, France

**Keywords:** community detection, natural language processing, social network, classification validation, social community, training without labels

## Abstract

**Introduction:**

We propose a hybrid methodology to evaluate the alignment between structural communities inferred from interaction networks and the linguistic coherence of users' textual production in online social networks. Understanding whether community structure reflects language use allows for a more nuanced validation of Community Detection Algorithms (CDAs) beyond assuming their outputs as ground truth.

**Methods:**

Using Twitter data on climate change discussions, we compare several CDAs by training Natural Language Processing Classification Algorithms (NLPCA), such as BERTweet-based models, on the communities they generate. Classification accuracy serves as a proxy for the semantic coherence of CDA-induced groups. This comparative scoring approach offers a self-consistent framework for evaluating CDA performance without requiring manually annotated labels. We also introduce a coverage–precision trade-off metric to assess community-level performance.

**Results:**

Our results show that the best CDA/NLPCA combinations predict a user's community with over 85% accuracy using only three short sentences. This demonstrates a strong alignment between structural and linguistic patterns in online discourse.

**Discussion:**

Our framework enables scoring CDAs based on semantic predictability and allows prediction of community membership from minimal textual input. It offers practical benefits, such as providing proxy labels for low-supervision NLP tasks, and is adaptable to other social platforms. Limitations include potential noise in CDA-generated labels but the approach offers a generalizable method for evaluating CDA performance and the coherence of online social groups.

## 1 Introduction

Online social networks have become central to the formation and expression of social identities. A growing literature investigates how individuals textual productions reflect their group affiliations and personal traits (Golbeck et al., 2011; Gosling et al., 2011). At the core of this inquiry is the assumption that group membership shapes both interaction patterns and language use.

Sociological and psychological theories support this dual structure. Identity Theory (Stryker, 1980; Stryker and Burke, 2000) posits that individuals align with groups through shared self-categorizations, while Bond Attachment Theory (Lawler et al., 2009) emphasizes group cohesion through repeated social interactions. Discourse theories (Gee, 1999; Wortham, 2001) highlight how linguistic practices signal social belonging.

In computational social science, these perspectives are modeled through networks of social interactions (e.g., retweets, replies) and textual data (e.g., tweets). Graph-based representations of social systems allow researchers to infer higher-order structures using Community Detection Algorithms (CDA). These communities, defined as subgraphs with dense internal links—are widely used to represent latent social groupings. Yet, the relationship between structurally inferred communities and the semantic coherence of their members' textual output remains underexplored.

Previous work has attempted to integrate text into Community Detection through hybrid models. Topic modeling techniques like LDA (Blei et al., 2003), embedding-based approaches (Mikolov et al., 2013), and BERTopic (Grootendorst, 2022) capture textual similarity to infer Community Structure. Conversely, some studies use linguistic metadata to refine structural clustering (Yang and Leskovec, 2015; Glavas and Vulic, 2019; Ribeiro and et al., 2021). While these approaches implicitly assume alignment between language and network structure, they rarely validate the correspondence explicitly.

In this study, we address this gap by proposing a methodology to evaluate the semantic coherence of CDA-derived communities using supervised NLP classification. Rather than treating CDA outputs as definitive, we use the classification accuracy of an NLPCA trained on each CDAs labels as a comparative measure of community coherence. The key idea is that if a CDA yields communities aligned with shared linguistic identity, then users texts should predict their community membership with high accuracy.

This comparative scoring approach offers a self-consistent framework for evaluating Community Detection performance without relying on externally annotated ground-truth labels. By testing whether linguistic content aligns with structurally inferred communities, we can assess the internal consistency of each CDA in capturing socially meaningful groups. We do not claim CDA labels represent absolute ground truth. Instead, our NLPCA performance metric reflects the degree of alignment between structural and discursive group formation. This allows us to rank CDAs according to how well they capture socially meaningful communities.

We apply this methodology to a Twitter dataset centered on climate change discourse. We compare the output of several CDAs, including Louvain, Infomap, and BEC, by training ensemble NLP classifiers on users tweets labeled by each CDA. The best-performing combinations achieve over 85% classification accuracy using only three short sentences from a random user.

A second key contribution of our analysis is to demonstrate that textual-based classification alone can be used to reconstruct social group membership with high fidelity, once trained on interaction-based communities. Unlike previous studies (Frank et al., 2018; Van Hee et al., 2018; Schwartz et al., 2013; Ferrara et al., 2014; Le Mens and Vedres, 2019), which apply NLPCA independently of social graph structure, we fine-tune classifiers on communities produced by CDA. This reveals not only which CDAs yield more linguistically coherent groupings, but also provides a tool for inferring user community membership solely from their language.

The objective of this study is thus twofold: first, to offer a scoring method that enables comparison between different CDAs based on their linguistic coherence; and second, to provide a pipeline for predicting community affiliation from text. Our approach integrates unsupervised Community Detection and supervised language classification into a unified validation framework. The key results show that this framework achieves high predictive accuracy and discriminative power across competing CDA models, using minimal text input. One limitation of the current work is that, in order to ensure sufficient training data per class, we reduce the number of communities to four dominant groups plus one residual class aggregating the remaining users. This reduces granularity but ensures model reliability. A second limitation is that, if a new Community Structure emerges over time, the training would need to be reperformed. However, this is not a major constraint in contexts where new training data is abundant, such as large-scale social platforms. Overall, our findings underscore the methodological and theoretical merit of combining structural and textual cues to define and validate online social groups.

This paper is organized as follows. Section 2 introduces the social network considered for this study, the CDA cases selected to illustrate our analysis, as well as some fundamental concepts of natural language processing. Section 3 describes the dataset and experimental setup. Sections 4, 5 present the evaluation of CDAs using NLPCA scoring. Section 6 discusses the broader implications of our approach, Section 7 outlines the main limitations and future work, and Section 8 concludes.

## 2 Communities in the Twitter social network

### 2.1 Climate change related tweets

We used the data from the Climatoscope project (Chavalarias et al., 2023) to extract the retweet network of online Twitter (now “X”) discussions about climate change over the year 2022. The Climatoscope project used Twitter's track API, which allowed to capture all tweets mentioning a given expression, collecting tweets based on a list of several dozens of English and French keywords related to climate change. This data collection was not exhaustive but represents a sufficiently large and diverse sample of climate change Twitter debates to understand the diversity of the social groups involved in them. Over the year 2022, 57M tweets have been collected, 32.1M of them being retweets.

We computed the retweet network, from 2022-07-01 until 2022-10-30, where the weight of an edge between two accounts equals the maximum number of retweets in either direction. The resulting network, weighted and undirected such that it can be processed by most CDA, was made of roughly 226,000 nodes and 430,000 edges. To identify the English speaking communities, we ran a standard Louvain Community Detection (Blondel et al., 2008) on this graph. We removed loosely connected nodes with degree strictly lower than 3, and kept the largest English-speaking communities only, pro-climate, and denialist.

**Figure 1:**
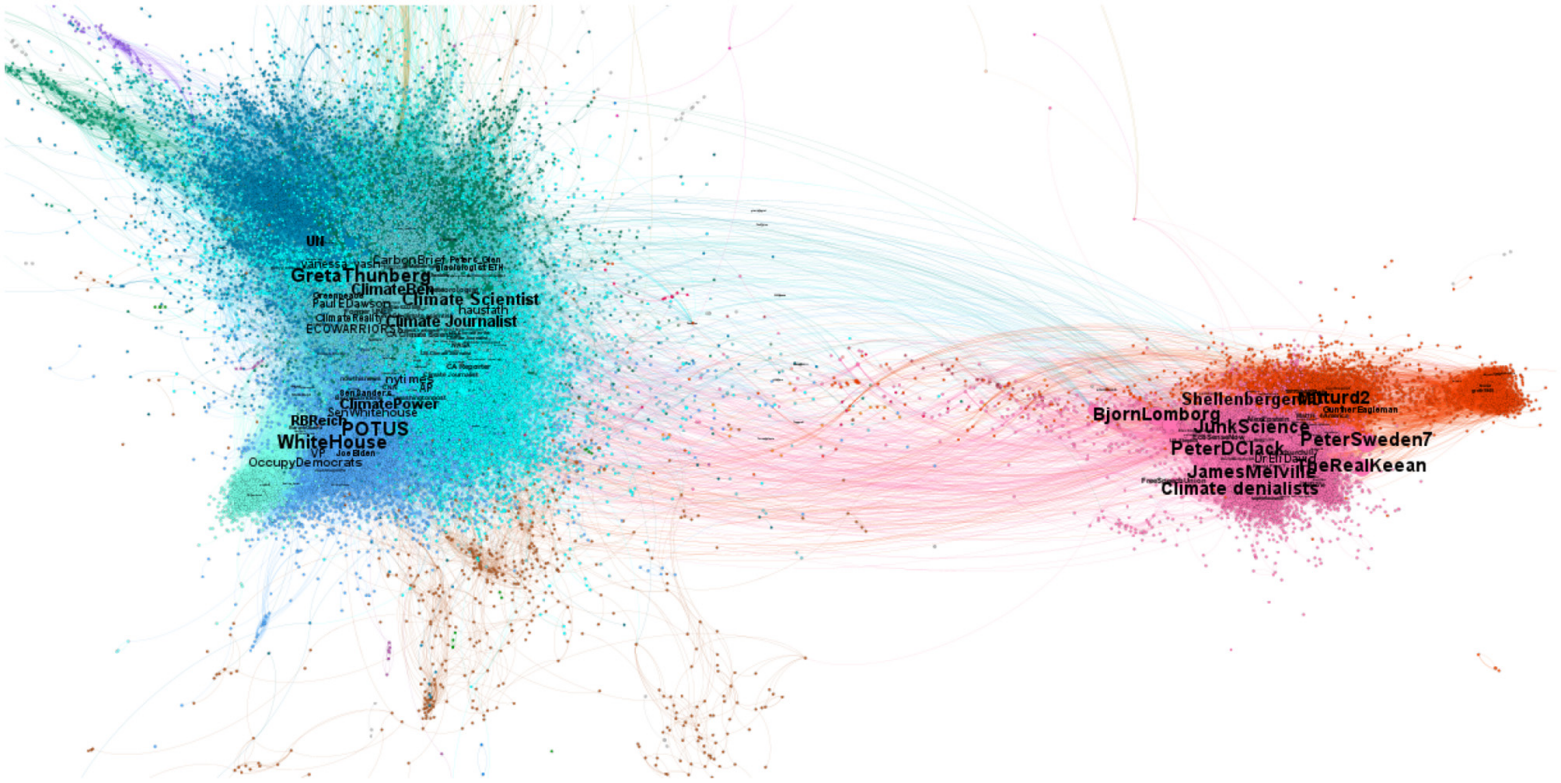
Visualization of climate change related tweets from 2022-07-01 until 2022-10-30, where colors represent different communities: cold/warm colors correspond to pro-climate/denialist users respectively. In total there are 29,347 accounts (nodes) and 361,559 retweets (edges) among those accounts.

The resulting graph was made of 30,000 nodes and 362,000 links. On one hand, these included international organizations (UN, COPX, UNICEF, NASA, etc.), climate activists (Greta Thunberg, Greenpeace, etc.) and communities centered on US Democrats: the left wing of the Democratic Party - around Bernie Sanders and Alexandria Ocasio-Cortez - and the mainstream Democratic party around Joe Biden, Kamala Harris and Barack Obama. On the other hand, the denialist communities feature Donald Trump's supporters and “Make America Great Again” (MAGA) Republicans, accompanied by other right-wing political leaders such as those of the United Kingdom Independence Party, and communities of influencer “experts” in climate science, who have their own audience and are densely connected to each other. It is in this latter denialist community that one finds accounts like JunkScience (Steve Milloy) notoriously supported by the fossil fuel industries such as the Heart Land Institute or the Competitive Enterprise Institute. In Figure 1, we show the resulting network using (Jacomy et al., 2014) for the spatial visualization. This algorithm employs a force-directed layout that minimizes the distance between nodes with strong connections and increases the distance between nodes with weaker connections. Visually, we observe the formation of two distinct clusters: on one hand, nodes in cool colors for which central nodes correspond to pro-climate users, on the other hand, nodes in warm colors where central nodes correspond to climate change denialists. Colors are based on the Louvain CDA (Blondel et al., 2008), which gives an initial overview of the network structure.

Our network will be make publicly available, with user IDs anonymized after referee reviews.

### 2.2 Community detection algorithms

In what follows, we demonstrate how NLPCA can serve as a “ground-truth” reference for Community Structure, allowing us to systematically evaluate the performance of different CDA methods. We illustrate this approach by comparing the results of three well-known CDA, providing a clear assessment of their alignment with textual based classification of social groups.

#### 2.2.1 The Louvain algorithm

The Louvain CDA is a method to extract non-overlapping communities from large networks (Blondel et al., 2008). It runs in time *O*[*n*·log*n*] where n is the number of nodes in the network. In the Louvain method, small communities are found by optimizing modularity locally on all nodes, then each small community is grouped into one node and the first step is repeated, where the modularity is the difference between the number of edges between nodes in a community and the expected number of such edges in a random graph with the same degree sequence (Newman and Girvan, 2004). It is defined as a value in the range [−1/2, 1],


$$\begin{array}{l} Q=\overset{m}{\underset{i=1}{\sum }}\frac{w_{ii}}{w}-\frac{w_{i}^{\text{in}}w_{i}^{\text{out}}}{w^{2}}. \end{array}$$ (1)

Here *w*_*ii*_ is the total weight of links starting and ending in module *i*, $w_{i}^{\text{in}}$ and $w_{i}^{\text{out}}$ the total in- and out-weight of links in module *i*, and *w* the total weight of all links in the network. To estimate the Community Structure in a network, Equation 1 is maximized over all possible assignments of nodes into any number *m* of modules. In (Lambiotte et al. 2014), a stability criterion of a network partition is introduced, a measure of its quality defined in terms of the statistical properties of a dynamical process taking place on the graph. The time-scale of the process acts as an intrinsic parameter that uncovers Community Structures at different resolutions. This method has been applied to find multi-scale partitions in the Louvain algorithm with a scale that we refer as “c” in what follows.

#### 2.2.2 BEC

(Gaume et al. 2024) propose a clustering method based on the optimization of the precision and recall (F-score) of a clustering relative to its ability to classify the edges of a network into clusters. It runs as an agglomeration process that reviews each edge of a network only once and merges the clusters of their nodes if this operation does not decrease the F-score. Hence there is a natural scale that is introduced, *s* which corresponds to the trade-off between precision and recall. It runs in time ~*O*[|*E*|] where |*E*| is the number of edges in the network.

#### 2.2.3 Infomap

Infomap reveals Community Structure in weighted and directed networks. The method decomposes a network into modules by optimally compressing a description of information flows on the network (Rosvall and Bergstrom, 2008). It is a two-level description that allows to describe the path of a random walk visiting nodes, using fewer bits than a one-level description. Basically when a walk is within a module (cluster of nodes), it spends long periods of time there. To optimize the compression, Infomap uses the map equation *L*(*M*) which gives the average number of bits per step that it takes to describe an infinite random walk on a network partitioned according to *M*:


$$\begin{array}{l} L\left(M\right)=q_{↷}H\left(Q\right)+\overset{m}{\underset{i=1}{\sum }}p_{↻}^{i}H\left(P^{i}\right) \end{array}$$ (2)

where M is a module partition among *m* modules. The first term corresponds to the entropy of the movements between modules and the second is the entropy of movements within modules. Each entropy is weighted, with *q*_↷_ being the probability that the random walk switches modules on any given step and $p_{↻}^{i}$ the fraction of intra-module movements occurring in module *i*, plus the probability of exiting module *i* such that $\overset{m}{\underset{i=1}{\sum }}p_{↻}^{i}=1+q_{↷}$.

The running time of Infomap depends on several factors, including the size of the network (number of nodes and edges) and the structure of the network (such as the density and distribution of edges).

### 2.3 Assigning tweets to categories

#### 2.3.1 Natural language processing

Natural Language Processing is one of the key pillars of artificial intelligence that enable to understand, interpret, and generate human language in an automated way. NLP algorithms are designed to understand and interpret the meaning of text data by mapping text into high dimensional mathematical vectors (this mapping is refereed as the embedding). If two vectors are close to one another in this space, it means that the two words or sentences are closely related. In the state of the art of NLP, this mapping relies on machine learning algorithms (see Achitouv et al., 2023 for a summary of the NLP techniques over the last decade), including transformer models (Vaswani et al., 2017), to better learn latent semantic links between words in a sentence. Bidirectional Encoder Representations from Transformers (BERT), is a natural language processing method based on the transformer architecture (Devlin et al., 2018). It represents a significant advancement in the field of language understanding and has been widely adopted for various language-related tasks as it is designed to capture contextual information from both the left and right context of words in a sentence. BERT can be fine-tuned for specific tasks, such as sentiment analysis, question answering, or named entity recognition. This fine-tuning process adapts the model to more specialized tasks and datasets. For this analysis we use the freely available BERTweet model (Nguyen et al., 2020) which is a fine-tuned model of BERT trained using a large corpus of tweets. Thus, we use BERTweet applied to our textual data, which generates high-dimensional representations of tweets that effectively capture Twitter-specific linguistic patterns, including hashtags, mentions, and emoticons. These embeddings serve as the input for our classification task.

#### 2.3.2 The classification algorithms

In order to classify the tweets into categories (selected communities), the BERTtweet embeddings are passed as input to four supervised classification algorithms, each selected for its complementary strengths in text classification:

- (a) Linear Classifiers with Stochastic Gradient Descent (SGD) (Bottou, 2010): Efficient for high-dimensional data and effective for linearly separable features.
- b) Support Vector Classification (SVC) (Cortes and Vapnik, 1995): Maximizes the decision boundary for improved generalization.
- (c) Multi-layer Perceptron Classifier (MLPC) (Rumelhart et al., 1986): A feedforward neural network that learns nonlinear feature interactions, using ReLU activation to introduce non-linearity and improve convergence stability. We use a small hidden layer structure (5,2) to balance expressiveness and computational efficiency.
- (d) Random Forest Classifier (RFC) (Breiman, 2001): A robust ensemble of decision trees that mitigates overfitting and improves classification robustness.

#### 2.3.3 The weighted ensemble model

Rather than relying on a single classifier, we use an ensemble approach to combine predictions from all four models, reducing variance and improving overall accuracy. Each model is assigned a weight based on its performance during validation: SGD & SVC contribute equally (*w*_*a*_ = 1, *w*_*b*_ = 1). RFC is weighted higher (*w*_*d*_ = 2) due to its strong individual performance. MLPC receives the highest weight (*w*_*c*_ = 3) as it captures complex nonlinear relationships effectively. The final prediction for each tweet is determined by a majority vote, where the category with the highest weighted count is assigned as the classification result. This hybrid approach allows us to assess how well different classification techniques align with Community Structures inferred from interaction-based clustering, providing a robust evaluation of textual coherence within social groups.

## 3 Methodology

In what follows every node is a user and every weighted edge corresponds to the number of tweets user *i* has re-tweeted from user *j*. For every CDA under review, we convert the directed network to an undirected graph and proceed as follows:

### 3.1 Step 1: attribution of a CDA categories

We run the CDA on the network resulting in N Community Structures that we map to *N*_*cut*_<*N*. To do so we keep the first *N*_*cut*_−1 communities with the largest number of users and we assign all other users to community *i* = *N*_*cut*_. This first step is essential for two reasons. First, the classification algorithms require a minimum size for the training set to perform accurate classification. When there are not enough user in a community, the number of tweets is too low. Second, depending on the parameters of the CDA, we generally have a number of communities that varies from a few hundreds to a few thousands (the limit being the number of nodes). These large numbers are not what a human interpretation of the community can handle if one is interested in classifying opinions of users. For instance in the climate tweets some communities can be climate denialists, pro-climate activists, pro-climate scientists, denialists advocating for fuel energy, etc. Larger numbers of communities with only a couple of users each are not particularly interesting to understand group dynamics. Hence the last category *i* = *Ncut* is a “catch-all term” one and is not used later on to evaluate the accuracy of the predictions. Importantly, this reduction to *N*_*cut*_ categories is applied uniformly across all CDA outputs, ensuring that all algorithms are evaluated under the same classification constraints. This design enables a fair and controlled comparison of their semantic coherence while maintaining sufficient data per class for reliable training.

### 3.2 Step 2: splitting users into training, testing lists

For each user we have its category attribution from the CDA. In order to run a ML classification on tweets, we split users into training and testing sets. For the training data we consider a fraction of users that corresponds to the most influential people. All the others are assigned to the testing set. This is motivated by (a) the fact that we don't need CDA to identify the most famous users (anchors) in a social network and (b) anchor tweets are often retweeted by many users, hence performing a ML classification on these tweets can help us find the community a random user belongs to. To select the most influential users, we could use a few metrics (page-rank, eigenvector centrality, degree centrality, etc.) or select by hand users that we believe have representative ideas. In what follows we consider influential people as users with an eigencentrality greater than 0.75-quantile, which is a measure of the influence of a node in a connected network (Shaw, 1954; Bottou, 2010).

**Figure 2:**
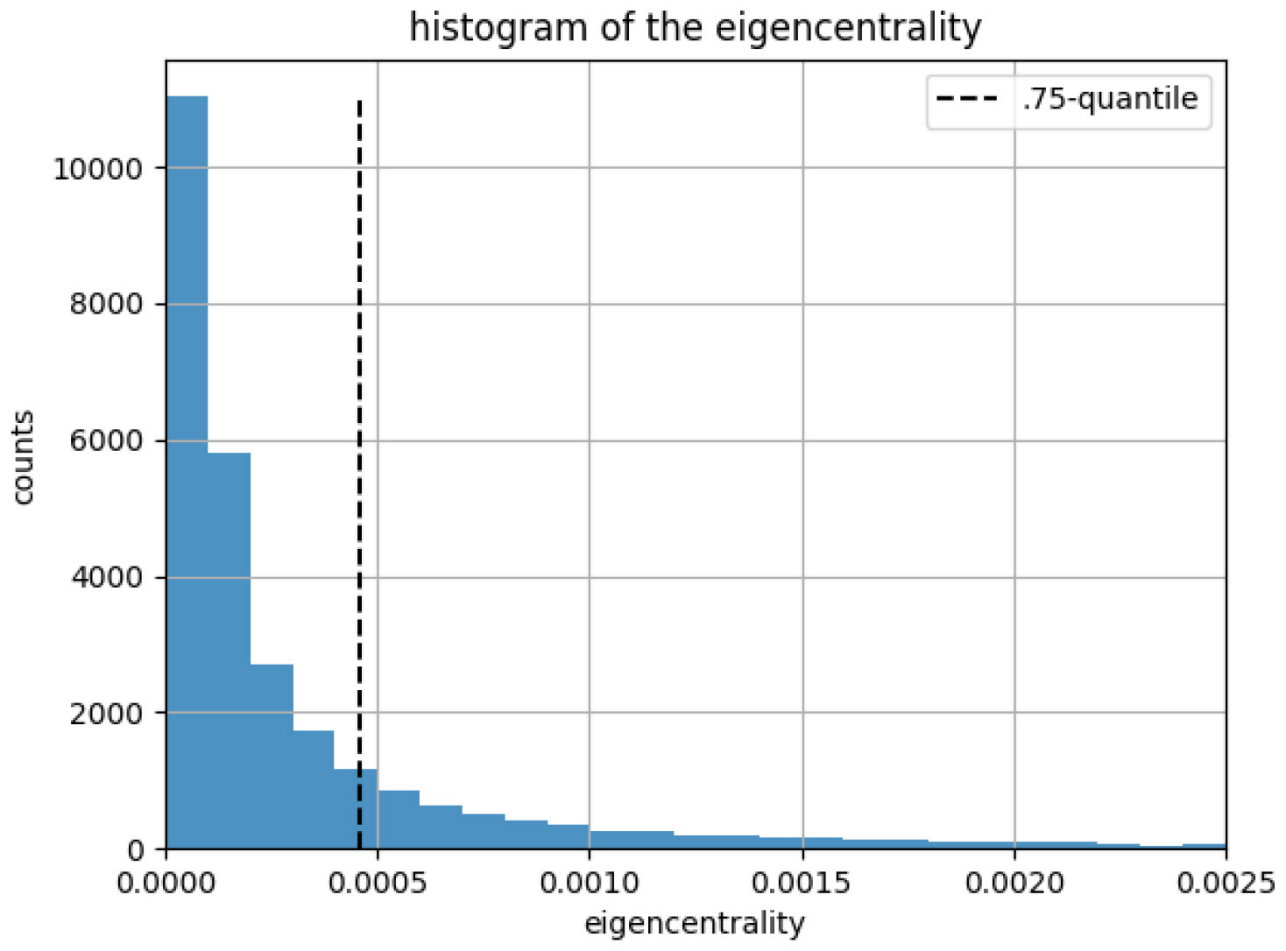
Histogram of user eigencentrality in our network. The vertical line corresponds to the 0.75-quantile, which corresponds to the cut between anchors and tested users.

Figure 2 displays the histogram of user eigencentrality. In social networks, power-law distributions are often associated with degree distributions, where a few nodes (users) have significantly more connections than others. However, eigenvector centrality takes into account not just the number of connections but also the importance of those connections. The vertical line corresponds to the 0.75-quantile. Users on the right-hand side are selected as anchors for training and users on the left-hand side are used to perform the testing.

We end up with 7,330 users (out of 29,000) for the anchors (training set) and the other users are assigned to the testing set. At the end we obtain 1,467,399 tweets from the anchors (for the training set) and 1,948,232 tweets from the other users (for the testing set).^1^ Then we select every tweet of all anchors flagged into *i*∈[1, *N*_*cut*_] categories in our training sets such that every tweet has a category associated to it, given by the category of the anchor who emitted it (identified in Step 1 above).

Note that this setup differs from a standard random training-test split, as it deliberately introduces a distributional shift between training and testing data. Rather than measuring in-distribution accuracy, our approach evaluates the models ability to generalize from highly influential users to the broader network, making it more akin to a transfer-learning framework. This distinction is important, as it aligns with our goal of leveraging well-defined community representatives to infer the likely community of less prominent users based on their textual production.

### 3.3 Step 3: performing a NLPCA classification training

For each CDA model we consider, we perform supervised NLPCA classification training (on each classification algorithm). We then apply the weighted ensemble model to the testing tweets to determine their textual category, which, in principle, should match the CDA category—assuming the entangled nature of the dynamical interactions of social network communities and their textual production. To recall, we use this assumption as a new metric for scoring the CDA algorithms.

In order to have unbiased training and testing datasets, given a CDA we select a fixed number of tweets per category_*i*_: $N_{train}^{i}$ for *i*∈[1, *N*_*cut*_]. For instance, we find that for *N*_*cut*_ = 5, we have obtained a convergence of the accuracy of the classification for $N_{train}^{i}=25,000$ tweets for the training sample of category *i*. We also select the same number of testing tweets for each category. Then we run the NLP classification algorithms described previously.

### 3.4 Step 4: evaluation of CDA performance

The final step is to evaluate each CDA classification of a user based on its agreement with the NLPCA. For each tested tweet we have both the category of the CDA and the category of the NLPCA. Each tweet is associated with a user, so we can reconstruct the NLPCA classification of the user using the k-tweets this user made in the testing set, with *k*∈[1, *N*] and *N* is an integer. His category *i* corresponds to the maximum count of his tweet flagged as *i* by NLPCA.

## 4 Global results

### 4.1 Can we precisely classify a random user?

**Figure 3:**
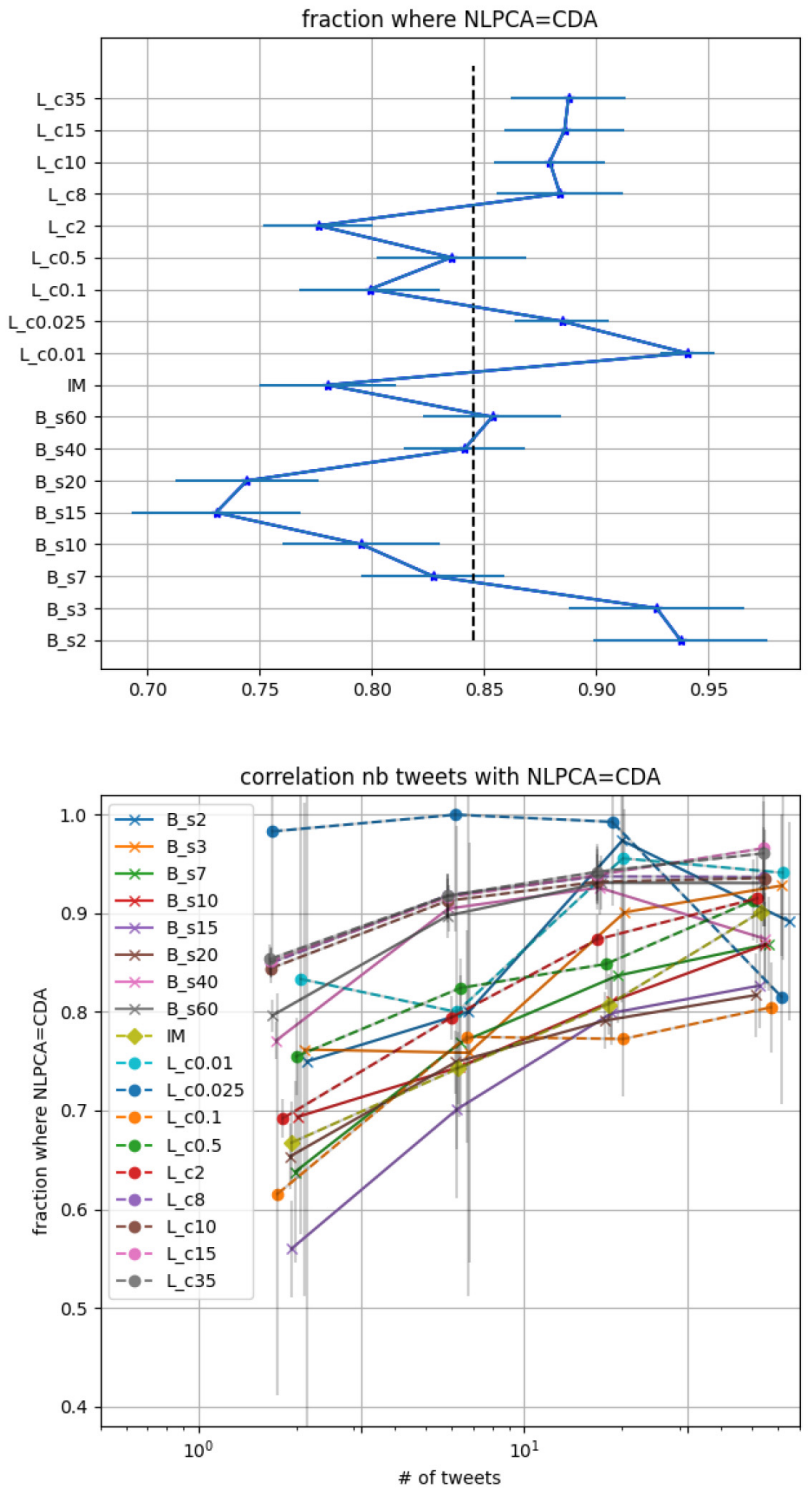
CDA accuracy based on its agreement with the NLPCA. *L*_*c*_ corresponds to Louvain with parameter c, *B*_*s*_ corresponds to BEC with parameter s and IM to Informap. **Top panel**: fraction of users where the NLPCA agrees with CDA regardless of the number of tweets. The error bars are 1-sigma deviation computed by Jackknife resampling. The vertical dotted line corresponds to the mean of the accuracy for all CDA we consider. **Lower panel**: fraction of user where the NLPCA agree with CDA as function of the number of tweet a user made in the testing set.

If one is interested in classifying a random user, the precision of the CDA needs to be privileged. In Figure 3 top panel we display the accuracy of the CDA based solely on the agreement with the NLPCA. Using the testing dataset with 25, 000 × *N*_*cut*_−1 = 100, 000 tweets we show on the left panel, for all CDA, the fraction of users that agrees with the NLPCA. The error bar corresponds to 1-sigma statistical deviation computed from a Jackknife resampling while the vertical dotted line corresponds to the average agreement using all CDA, here it is 85% agreement. We note that randomly assigning a category to each tweet would result in an expected agreement of 1/4, given our balanced testing set for each CDA.

From this figure, we deduce that the best re-scaled modularity parameters for the Louvain correspond to *c* < 0.025 while the optimal parameter for the BEC is *b* < 7. For these parameters the CDA classifications agree with the NLPCA at a precision >90%, remarkably.

On the lower panel of Figure 3, we display the fraction of user where the NLPCA agrees with the CDA as a function of the number of tweets made by users in the testing set. The binning of tweets number is logarithmic. The first bin corresponds to [1, 3] tweets, second to a number between [4, 10], third to [11, 31], and then ≥32 tweets. As expected, it is more challenging to classify a user based on a few tweets compared to a larger number of tweets. However it is still quite impressive to see an agreement at ~85% for several CDA, considering that [1, 3] tweets can characterize a randomly selected user. Then we observe that the agreement increases with the number of tweets. The decrease of some curves is not statistically significant as the number of users who posted more than 15 tweets reduces to less than 10 in some cases. Poisson errors are displayed in light gray on the figure.

### 4.2 Precision vs. coverage: can we categorize most users?

A key point to address in the CDA is the number of users covered by our selected categories. Indeed, when the percentage of users in our selected categories is low, one might doubt the utility of communities toward understanding the opinion of most users.

**Figure 4:**
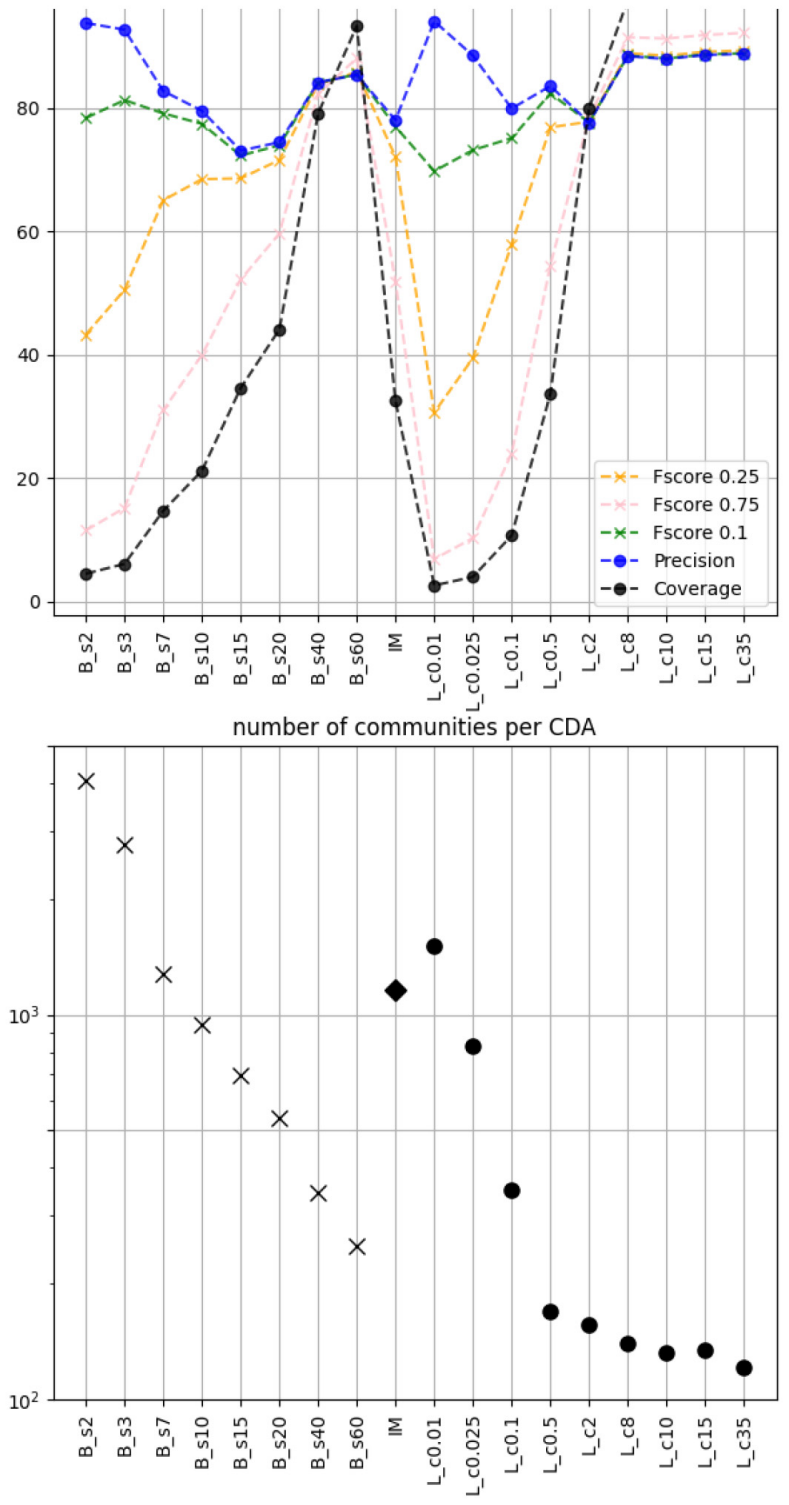
**Lower panel**: number of communities found by the CDA. **Top panel**: Precision (percentage of agreement between CDA and NLPCA categorization), Coverage (percentage of users covered by our 4 selected categories), and F-score (weighted score between precision and coverage).

In Figure 4 lower panel we show the number of identified communities per CDA. Depending on the algorithm and on the parameters (if any), the number of communities can change by an order of magnitude. Hence the 4 selected (biggest) communities for each algorithm range from a few per cent of the total number of users, to most of them. This is what we refer to as the coverage (black curve) in the top panel of Figure 4. This coverage can be compared with the *precision* we previously considered (percentage of agreement between CDA and NLPCA in the classification of test set users). As one can expect, when the coverage is low, the precision is high because the textual production within small communities is not diverse. The precision decreases as the coverage increases, until a minimum is reached. Then we see the opposite trend for Louvain and BEC: precision and coverage increase together.

For instance, if one is interested in categorizing 80% of users with a precision of 90% then we see that the best option is to use Louvain with parameter *c*≥8.

For group analysis in social science, the coverage of the users is most certainly of primary importance hence a good and natural score to use is the F-score,


$$\begin{array}{l} F_{\beta }=\left(1+\beta^{2}\right)\frac{P\times R}{\beta^{2}P+R} \end{array}$$ (3)

where *R* is the recall, here corresponding to the coverage, *P* the precision, and β is a real factor chosen such that the recall is considered β times as important as the precision. In Figure 4 top panel we display the F-score function weighting the coverage at 0.1, 0.25, and 0.75. In such a case it is clear that the best performing CDA corresponds to the Louvain, with a best re-scaled modularity parameter *c*≥8, while the optimal scale s for BEC is ≥40 (maximum of all F-scores).

The number of categories identified by the CDA is also something interesting to consider. In Figure 4 lower panel we see that the number of communities decreases when the coverage increases. Interestingly, there is a case where BEC, Infomap and Louvain have approximately the same number of communities: ($B_{s}^{7};IM;L_{c}^{0.01}$). For this triplet, the Coverage is (15%; 33%; 3%) while the Precision is (83%; 78%; 94%). This means that for Infomap we have a bigger clusters than for the Louvain, BEC being in between. Another interesting triplet is ($B_{s}^{7}$; *IM*; $L_{c}^{2}$) for which the F-score with weight 0.1 is similar and about ~80%. Finally, for ($B_{s}^{15}$; *IM*; $L_{c}^{0.5}$) the coverage is the same but the precision (73%; 78%; 82%) shows that Louvain provides a better choice.

### 4.3 A Pseudo-Entropy measure of the NLPCA

Interestingly we may characterize the average number of distinct NLPCA categories for a user based on all his tweets. For instance if a user made 10 tweets, the NLPCA can assigns the 10 tweets to his CDA category, e.g. category 1, but it can also assign 5 tweets to category 1, 2 tweets to category 2 and 3, and 1 tweet to category 4. This provides a measure of the entropy of the categorization that we test for all CDA. If the entropy is null then it means that the NLP categorization of a user is without a doubt in the CDA category.

**Figure 5:**
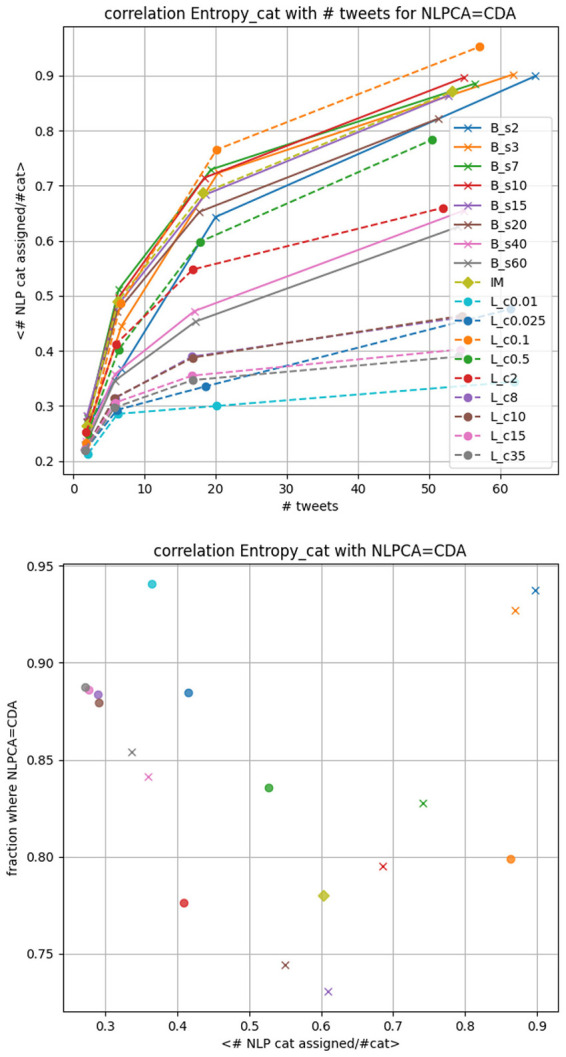
correlation between the entropy measure and the number of tweets **(top panel)** and the classification agreement with NLPCA **(lower panel)**.

In Figure 5 we display on the top panel the average of this entropy over all tested users as a function of their number of tweets. The more tweets a user has posted, the more likely it becomes for the NLPCA to assign a tweet to a different category than his CDA category. So for a fixed number of tweets we can compare the entropy of the different CDA. On the lower panel we display the fraction of users where the NLPCA categorization agrees with the CDA, as a function of this entropy measure.

Interestingly, the Louvain algorithm is the CDA algorithm that leads to the more stable NLPCA (users are assigned to a fewer number of categories) compared to the BEC CDA, while Infomap lies in the middle. This could be interpreted as a more subtle Community Structure in the BEC, where users are not necessarily central in their own community. This intuition is also confirmed by the coverage of users the CDA find. For BEC, the entropy is clearly related to the coverage of users: the entropy decreases as coverage increases. For the Louvain, this entropy is stable for *c*≥8, similar to what we find for the coverage in Figure 4, while it increases when the coverage ≤ 80% which corresponds to *c* < 8.

Again, for the triplets case we previously considered with a similar number of communities ($B_{s}^{7};IM;L_{c}^{0.01}$), ($B_{s}^{7};IM;L_{c}^{2}$) where we have a similar F-score and ($B_{s}^{15}$; *IM*; $L_{c}^{0.5}$) where the coverage is the same, we observe that the entropy among these CDA is significantly different, suggesting different community properties for each CDA.

Finally, we have checked that the wrongly assigned users do not show distinguishable global properties on the network. For each CDA, we compare the distribution of the vector centrality of the wrongly assigned users to the distribution of all users without finding significant deviations. This is also true when comparing the CDA among them because the test set of users is mostly the same for all CDA. Among the two best performing CDA: $B_{s}^{60}$ and $L_{c}^{10}$ we find the 15% and the 12% of wrongly assigned users in both CDA (respectively), are about half the same users. This category of users are either “indecisive” or easily influenced, and can be an interesting social group to study in their own right.

## 5 Social analysis of the communities for the CDA in the light of NLP

We now turn to a more refined analysis of the CDA categories by flagging influential users that belong to them. Those are the users from the training set that are public figures, or represent political parties, media, or are influencers. We also report a few users that have tens to hundreds of thousands of followers with a strong view on climate change. Among the pro-climate users we have *CleanAirMoms* 37,000 followers, a community of “moms and dads who are uniting for clean air and our kids' health”; *wtpBLUE*, 12,000 followers, and a “grassroots GOTV organization dedicated to electing Democrats.” Among the American denialists we select *MattH*_4_*America*, 100,000 followers defined as “America First - Patriot”; *Catturd*, 2.5M followers, a MAGA influencer; *DrEliDavid*, 620,000 followers, entrepreneurs; for the Australian denialists: *AlexandraMarshall* 77,300 followers, writer/artist; *PeterDClarck*, 37,000 followers, journalist advocating for carbon emission. In the Canadian denialists we have: *GasPriceWizard*, 54,000 followers, a former liberal MP; *Sunlorrie*, 108,000 followers, journalist; *TheRealKeean*, 300,000 followers, a journalist.

### 5.1 Community structures for the same coverage

**Figure 6:**
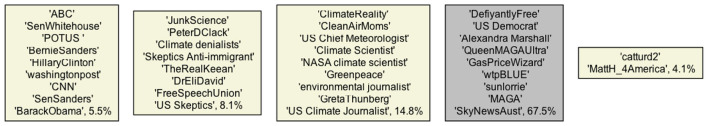
Infomap Community Structures for the selected users. Yellow boxes correspond to one of our four categories while the gray box correspond to the catch-all-term category. The percentage in each box corresponds to the fraction of all users in each of the category.

Infomap does not provide a scale parameter, the four biggest communities cover 32.5% of all users. These communities, as displayed in Figure 6, are easy to interpret. The largest (14.8%) corresponds to pro-climate activists, scientist and media. The second (8.1%) corresponds to climate denialists that focus on denying that CO_2_ and fossil fuel are the cause of climate change. The third (5.5%) corresponds to Democrats and some mainstream media, while the fourth (4.1%) corresponds to communities around MAGA influencers for whom climate change is a“hoax” or a“cult.”

We can compare these communities to what Louvain and BEC provide for about the same coverage (for *c* = 0.5 and *s* = 15, respectively). Interestingly, they differ in the way they categorize the top influencers. On the denialist side, Infomap, and BEC tend to agree on the cluster of clear MAGA supporter and on another more dedicated to “experts” and lobbies, while Louvain places some big influencer from the lobbies cluster into the MAGA cluster.

The situation is different for the pro-climate communities. Here, Infomap and Louvain agree to make a environmental NGO/activits cluster and another with Democrat leaders such as Biden, Obama, Hilary Clinton, and Bernie Sanders, while BEC includes Bernie Sanders in the environmental NGO/activits cluster. Both clustering make sense but we might argue that from the point of view of the climate debate, Bernie Sanders was indeed closer to NGO/activits than the mainstream Democrats in his public statements, which is reflected in the BEC clustering.

From a narrative perspective, the precision provides a measure of how homogeneous the textual content of these communities is. For the same coverage, the Louvain provides the best precision (~82%), followed by Infomap (~78%) and BEC (~73%).

### 5.2 Community structure evolution for different parameters

**Figure 7:**
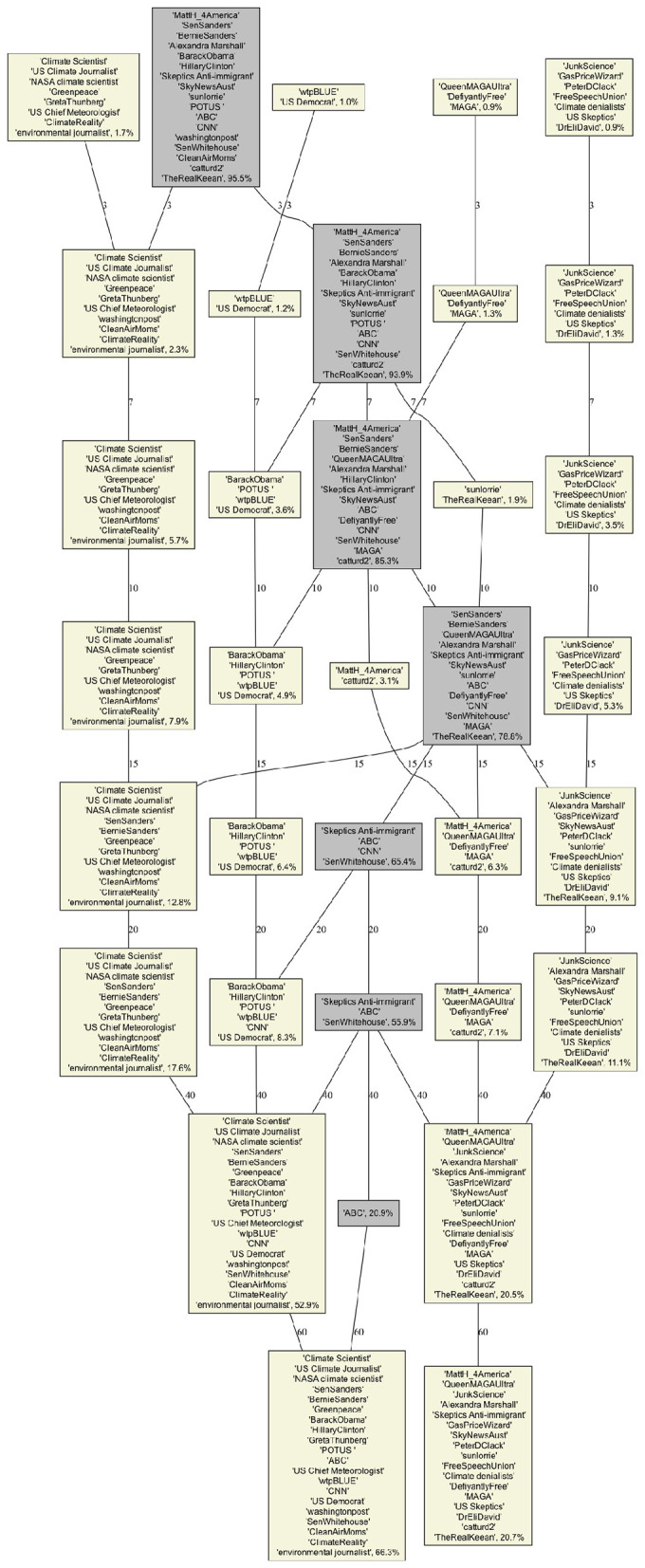
BEC Dendogram of our selected users community as function of the BEC parameters s (displayed on the edges). Yellow boxes correspond to one of our 4 categories while the gray box correspond to the catch-all-term category. The percentage in each box corresponds to the fraction of all users in each of the category.

**Figure 8:**
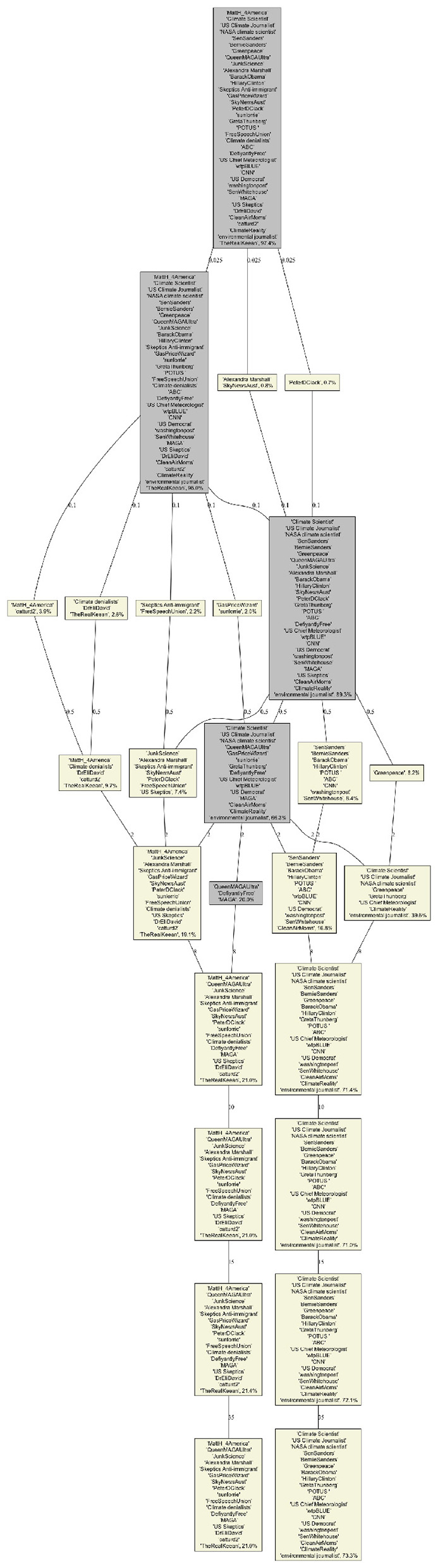
Louvain Dendogram of our selected users community as function of the rescaled modularity parameter c (displayed on the edges). Yellow boxes correspond to one of our 4 categories while the gray box correspond to the catch-all-term category. The percentage in each box corresponds to the fraction of all users in each of the category.

The analysis of the evolution of Community Structures as a function of the scale parameters *s* for BEC (Figure 7) and *c* for Louvain (Figure 8) provides more insight into these differences. Similarly to the coverage evolution, for small values of *s* or *c* we have many small communities, and hence most users belong to the catch-all-term category (displayed in gray). As the values of these parameters increase, the number of communities decreases and the fraction of users in our four categories increases. For our selection of users we observed that they merge into only two groups at high scales: *s*≥60 and *c*≥8, for BEC and Louvain respectively (the other two categories do not contain our selected influencers). These two groups are the same for the two CDA and can be interpreted as climate denialists and pro-climates. In these cases, the coverage >85% and the precision >85% for the two CDA becomes similar.

Interestingly, BEC identifies well the four categories of opinion among the top four communities even for low scale resolution (community sizes <1%) and integrates more actors as the scale is increased, Louvain focuses on peripheral communities and important ones appear only for medium scales (community sizes 2%−15%). This is not surprising because optimizing modularity leads to merging small communities into larger ones, even when those small communities are well defined and weakly connected to one another (Kumpula et al., 2007). Thus, we only see the four categories (given our selected users), when *c* = 0.1. Moreover, it seems that BEC faithfully reflects the structure of positions on climate, with an initial integration of Bernie Sanders' current into the community of pro-climate activists, followed by a merger of all pro-climates into a single community for high values of *s*.

## 6 Discussion

The study of social networks has experienced significant growth, leading to substantial advancements in understanding the dynamics of social structures and interactions (Borgatti and Halgin, 2011; Lazer et al., 2009; Newman, 2003). When analyzing social networks built upon social interactions, Community Detection Algorithms (CDAs) play a central role: they reduce the complexity of a large interaction network into higher-order structures, allowing us to study patterns at the community level.

Most CDAs identify communities based solely on network structure—e.g., link density—without considering whether users within a community share a coherent opinion or narrative. In this work, we build on the assumption that users in the same community often produce semantically related content. By leveraging this connection, we develop a new scoring framework to assess CDAs: rather than evaluating the Community Structure based on structural features alone, we measure the internal linguistic coherence of communities via a Natural Language Processing Classification Algorithm (NLPCA).

This hybrid framework reveals meaningful insights. First, it enables a fair comparison between different CDAs by scoring their output in terms of semantic predictability. This is particularly valuable given that most CDAs optimize different objective functions and are difficult to compare directly. Second, it allows for the identification of the most coherent Community Structure in a specific domain, which can guide further analysis and applications.

Moreover, our framework uncovers interesting boundary cases. By analyzing users who are misclassified by the NLPCA relative to their CDA assignment, we can identify individuals whose linguistic behavior diverges from their structural affiliation. These users—potentially bridges between communities—represent socially significant profiles for further study in the context of opinion dynamics.

Finally, although the NLPCA is trained on CDA-generated labels, its role is not merely to replicate those labels but to provide a scoring function for evaluating semantic coherence. This design ensures internal consistency across CDAs, and if new communities emerge in real-time applications, the model can be retrained using the same pipeline, provided that sufficient data is available.

## 7 Limitations and future work

A first limitation of this study lies in the size of the label space. To ensure that the supervised classifiers had enough data per class to train effectively, we retained only the four largest communities for each CDA and grouped the remaining users into a residual “catch-all” class. While this approach ensures training reliability and interpretability, it reduces granularity and excludes micro-communities. Future work could extend this methodology to larger label spaces, possibly by exploring few-shot or class-imbalance-aware techniques.

A second limitation is the assumption that the Community Structure remains fixed. In real-world dynamic social systems, new communities may emerge over time. In such cases, our methodology would require retraining the classifier on updated CDA outputs. However, this is not a major concern in data-rich environments such as large online platforms, where new training data can be collected continuously. Also note that if the initial CDA produces structurally incoherent or noisy communities, the NLP-based scoring could also be unreliable, potentially leading to misleading relative rankings. While our approach assumes a minimal level of structural and semantic validity in the CDA output, in the present case study this risk is not observed, as shown in Section 5, where the detected communities are found to be coherent and interpretable from a social science perspective.

Lastly, although our approach is not intended to discover new communities, it could be extended in future research to examine how semantically emergent subgroups relate to existing structural partitions, or to study cases of partial semantic overlap between structurally distinct communities.

## 8 Conclusion

This study proposes a novel framework for scoring Community Detection Algorithms by leveraging the coherence of users' textual production. By aligning network-based and discourse-based representations of social groups, we offer a method to score CDAs based on their semantic predictability.

A second key contribution of our work is the demonstration that NLP classifiers, once trained on CDA-labeled data, can predict user community membership with high accuracy using only a few sentences. This opens the door to lightweight, language-based classification of users in social networks, enabling applications ranging from opinion profiling to misinformation detection.

In addition to scoring CDAs, our approach offers practical benefits. For instance, a reliable CDA can serve as a proxy for community labels in low-supervision NLP scenarios, allowing for the fine-tuning of pre-trained models on unannotated data. This synergy between structure and content is particularly useful in contexts where manual labeling is not feasible.

Finally, while Twitter/X provided a well-structured case study, our method is highly adaptable to other online platforms. For example, in decentralized social networks such as Mastodon, one could apply this approach by analyzing the re-toot interaction network in combination with the content of toots. Similarly, Reddit discussion threads, Discord message networks, or Bluesky social graphs provide alternative settings where our method can be used to evaluate Community Structures and group narratives. This adaptability ensures that the approach remains relevant despite changes in social media ecosystems. While CDAs effectively capture some social structures, to the best of our knowledge, there is no generic method for assessing the nature of these social structures in terms of cultural characteristics such as semantic homogeneity. Our hybrid methodology, which combines network topology with textual production in the digital space, provides a general approach to scoring the differences among the CDAs communities. This allows a more refined analysis of on-line social groups.

## Data Availability

The datasets presented in this study can be found in online repositories. The names of the repository/repositories and accession number(s) can be found below: https://github.com/IxandraAchitouv/CDA_NLPCA.

## Footnotes

1 We note that the total number of anchors does not impact our analysis when this number is divided by at least 3 times. The only issue in reducing the number of anchors is that we obtained sometimes a smaller amount of tweets than our threshold for training a given category of a given CDA. This is particularly true when the number of communities is greater than a few thousand.
